# Prof. Millar on Tobacco

**Published:** 1861-03

**Authors:** 


*Prof. Millar on Tobacco.*—[This learned surgeon has of late been paying much attention to the social questions connected with the medical profession. We noticed a year ago his work "on Prostitution," and later still his work "on Alcohol its use and abuse." He has now turned his attention to To bacco, and as we have lately given the views of several authors on this important luxury, we extract from the *Edinburgh Scotsman* the report of Prof. Millar's speech at a recent meeting of the *British Anti-Tobacco Society.* The speeches of the learned opposers we do not trouble ourselves about, as their opinion is no weight outside their own city, and of precious little there.]

Professor Miller moved the first resolution:—"That as the constituent principles which tobacco contains are highly poisonous, the practises of smoking and snuffing tend in a variety of ways to injure the physical and mental constitution." (Hear, hear.) That statement, he said, perhaps required a little explanation. He did not mean to say that because a substance was in itself poisonous, its use in all possible circumstances must prove injurious to mind or body, or both together; but what he said was, that tobacco and other narcotics, being well known rank poisons, if they were taken unnecessarily, were taken unwisely and unsafely. This was just in accordance with one of the simplest and most certain rules in therapeutics —that if there was a diseased condition in a man's body which required the use of tobacco, or opium, or prussic acid—strongly poisonous as those remedies were—yet when judiciously and skilfully administered, they might be taken not only innocuously, but with the greatest positive advantage. They would do the man no harm, but good. All this was in accordance with what was technically called the law of tolerence; but this law had a converse; and the converse here was that if a person took a poison into his system which was not requiered by any diseased condition of body, there was no law of tolerance to protect him; and it not only did him no good, but it did him harm more or less. Now, tobacco is one of the strongest narcotic poisons with which we are acquainted; and the shape in which he would put the question was—were the people who used it so extensivcly protected by the law of tolerance, or were they met by the converse of that law, which rendered the use of it unsafe? He presumed that 99 out of every 100 of those who were to be seen going smoking about the streets, or using tobacco in some form or other, would never dream of putting in such a plea as that they used it medicinally for some disease under which they were laboring —therefore, it naturally and necessarily followed from the principle which he had laid down that all these people were injuring themselves more or less. (Hear, and applause,) He would next proceed to inquire what were the evil effects which tobacco produced upon the physical and mental constitution. Medical men were but too familiar with these evil effects. They knew that it injured the whole organism, put the stomach out of order, and weakened the frame. But it acted mainly upon the nervous system—not strengthening, on the contrary, weakening it and depriving it of its tone. No man who was a hard smoker had a steady hand; and as the ancients said, *Ex pede Herculem*, he would say, *Ex manu hominem*—by its effects on the man's hand we might judge of its effect upon his whole nervous system. But not only had it a debilitating and paralyzing effect; but he could tell of patients who were completely paralyzed in their limbs by inveterate smoking. He might tell of a patient of his who brought on an attack of paralysis by smoking, who was cured indeed by simple means enough, accompanied with the complete discontinuance of this practice; but who afterwards took to it again, and got a new attack of paralysis; and who could now play with himself as it were, because when he wanted a day's paralysis or an approach to it, he had nothing to do but to indulge more or less freely with the weed. Then as to the effect of smoking upon the intellectual powers—for he would leave its moral and social consequences to other speakers—there could be no doubt that it obfusticated mental vision more or less. "Blowing a cloud" was a very good symbol of the cloudy kind of ratiocination which the person who did so get into in consequence. It might not be easy to prove that in individual cases, but it was not difficult to do so on a large scale. Only the other day, the French—among whom the practice was carried even to a greater extent than with us—made an estimate of its effects in their schools and colleges. They took the young men attending these institutions, classified them into those who smoked habitually, and those who did not, and estimated their physical and intellectual standing—perhaps their moral standing too, but he could not say. The result was that they found that those who did not smoke were both the stronger lads and better scholars, were altogether more reputable people and more useful members of society, than those who habitually used the drug. What was the consequence? Louis Napoleon—one of the good things which he had done—instantly issued an edict that no smoking should be permitted in any school, college, or academy. This edict in one day put out about thirty thousand pipes in Paris alone. Let our young smokers put that in their pipe and smoke it. That was one of the advantages of an autocraacy; and he (Prof. Miller) would be very glad if the Lord Provost had the power to issue a similar edict in Edinburgh, which he was quite sure would derive much benefit from it. Perhaps there might be smokers present who thought this all stuff; and who were ready to say that they knew from experience that smoking did them good, and that they could reason a great deal better after a comfortable smoke than they could before it. Very good; but that only made his case all the stronger.

Let a healthy man, unaccustomed to smoking, take up a pipe of tobacco and smoke it to the end, or give him three or four pinches of snuff in succession, and then let them ask him whether he felt better or worse. Set him to work out a difficult problem, and see what was the condition of his reasoning powers. The man would at once admit that they were in very bad condition. The men who felt the better after using a narcotic were just the men who were slaves to it; and it was merely the disordered and morbid state of their physical and nervous system, which required to be tickled up again with that stimulant which had already exhausted and weakened them. Professor Miller went on to refer at some length to the effects of opium among the Chinese, and showing many points of similarity between it and tobacco smoking when extensively indulged in. He then referred to the increase of the use of tobacco, particularly in the form of smoking among the young, remarking that its prevalence—taking into account its unfailing physical, intellectual, and moral effects—was calculated to alarm those who had the welfare of the community at heart. After relating several anecdotes tending to show the extreme difficulty, and in some cases the impossibility, of giving up the habit when it had been fairly formed, notwithstanding the pain which it caused by inducing cancer or other diseases, Professor Miller went on to say it could not be denied that there was a diversity of medical opinion regarding the injurious effects of the use of tobacco. But he would ask them to inquire who were the medical men who would come forward as the champions of tobacco? They were men who were themselves smokers. But these men, by their own addic tion to this vice, had put themselves out of court, and their opinion was therefore of no consequence. The man best qualified to give an opinion on the subject was the man who had been so far enslaved by the habit, but who had given it up. Professor Miller went on to say that he was in this position, having at one time been addicted te snuffing, but after ten or a dozen vain and difficult attempts he had given it up. He had tried it both ways, and he had a bright recollection of the comforts of snuffing. The first pinch in the morning was delicious; the pinch after dinner was—magnificent! But until that pinch was got he was stupid, dull and restless, aud the effect, both mentally and corporeally, was evil, and nothing but evil.

Mr Reynolds, Secretary to the British Anti-Tobacco Society, seconded the resolution. He began by stating that tobacco contained two poisonous principles, one of which was nicotin, and the other an essential oil which was so injurious and acrid in its fumes that if a single drop of it were burned in the hall, every one of his auditors would feel themselves compelled to rush to the door. He proceeded to narrate a number of anecdotes which had come under his own observation and experience tending to show the injurious effects of "the weed" upon the physical and mental constitution of man, as well as the benefits which had followed the total discontinuance of the indulgence.—*Nashville Med. & Sur. Jour.*

